# Assessment of Gender Bias During Paramedic-Physician Handoffs

**DOI:** 10.7759/cureus.41709

**Published:** 2023-07-11

**Authors:** Katie Pettit, Chelsea Harris, Kathryn Smeltzer, Elisa J Sarmiento, John T Hall, Cody Howell, Mark Liao, Joseph Turner

**Affiliations:** 1 Emergency Medicine, Indiana University School of Medicine, Indianapolis, USA; 2 Emergency Medicine, SSM Health DePaul Hospital-St. Louis, Bridgeton, USA

**Keywords:** communication in healthcare, emergency medicine resident, safe patient handoff, gender bias, emergency medical service, pre-hospital emergency medicine

## Abstract

Objective

Gender bias against female physicians has been frequently demonstrated and associated with negative feelings toward their careers. Gender bias has also been demonstrated in prehospital clinical care. However, potential gender bias during paramedic-physician handoffs has not been studied. This study aimed to identify gender bias during interactions between prehospital personnel and emergency physicians at the time of patient handoff.

Methods

An observational study was conducted at an urban academic emergency department. Observers were trained to record information from paramedic-physician handoffs but were blind to the nature of the study. The primary outcome was to whom paramedics initially directed the focus of their handoff report based on physician gender, with secondary outcomes of to whom paramedics directed most of their report and whether they asked about further questions based on physician gender.

Results

There were 784 observed handoffs. There was no significant association between the gender of the physician and which physician received first attention (χ^2^ {1, N = 782} = 0.9736, p = 0.3238) or majority attention (χ^2^ {1, N = 780} = 1.9414, p = 0.1635). Paramedics were more likely to ask questions to male attendings than female attendings (χ2 {1, N = 784} = 4.4319, p = 0.0353).

Conclusion

We identified limited differences in communication based on gender between paramedics and physicians during emergency department patient handoffs.

## Introduction

Gender bias has been demonstrated in many areas of healthcare and affects not only patients but also faculty and resident physicians [1,2]. Female physicians experiencing gender bias are more likely to “leave medicine/retire early” and “not recommend their specialty to trainees or family members” [3]. For emergency medicine resident physicians, there is evidence of gender bias in evaluation from both faculty and nursing [4,5]. However, our current understanding of gender bias in healthcare settings is limited, as most studies on this topic come from the analysis of perceptions of interactions rather than direct observation.

In many clinical settings, gender bias manifests as microaggressions [6]. Microaggressions are brief, often commonplace insults, intentional or unintentional, that can have a harmful psychological impact on a person or group [7]. Their effects have been shown to be more pervasive and deleterious than previously thought. A recent study found underrepresented minority medical students who experience microaggressions have significant feelings of burnout, insecurity, and invalidation [8]. Female physicians report a high frequency of experiencing such microaggressions [9].

Bias manifested in the form of microaggressions could affect the quality of clinical communication. In the setting of a hectic emergency department, high-quality communication is essential, especially during the transfer of a patient from prehospital personnel to physicians. Patient handoffs are known to have significant safety implications and potential for error [10,11]. Paramedic handoffs have been studied in terms of their informational content [11,12]. However, the role of the gender and the potential presence of gender bias during interactions between pre-hospital and emergency department (ED) physicians has not been well studied. It is possible that gender bias could have an impact on communication during this important patient care activity.

Current research involving gender bias and prehospital personnel has focused primarily on clinical care, finding differences in the frequency of aspirin use and electrocardiograms in non-traumatic chest pain [13] or in transport time for patients with myocardial infarction [14]. Bias by prehospital personnel during interpersonal communication has received less attention. This study aimed to identify gender bias during communication between prehospital personnel and ED physicians during patient handoffs.

## Materials and methods

Study design and setting

An observational study was conducted at an urban academic emergency department with 90,000 visits annually. A convenience sample of paramedic-physician handoff interactions was observed between June 2021 and July 2021. Observations were done according to the availability of the data collectors, but observation times included each day of the week as well as morning, evening, and overnight periods. Eligible interactions included patients brought by EMS personnel into high-acuity rooms in the ED (a dedicated geographic area within the department) when both faculty and residents were present at the time of patient handoff. In our system, attendings and residents are generally distinguishable by scrub color, but prehospital personnel are not given routine instructions indicating that they should direct their reports to one or the other. Encounters involving patients with a primary psychiatric diagnosis and level 1 trauma activations were excluded.

This study was approved by the Indiana University IRB, protocol number 11140. Consent was waived as the study was observational and collected no identifying information on any subject.

Materials and data collection

Two observers (JH, CH) were trained in ED navigation and encounter enrollment by supervising investigators (JT, KS). Observers were told they would be collecting data during paramedic-physician handoffs but were led to believe that the primary objective of the study was to assess handoff content and were kept blind to the bias component of the study. They were instructed to read background material on handoff content [12] before their observations to reinforce this belief.

Data was collected on a standardized form and included the attending physician and resident physician for each encounter. The observer recorded whether the paramedic first directed their handoff presentation to the attending physician or the resident physician and whether most of the presentation was directed to the attending physician or the resident physician. Observers also recorded whether the paramedic asked if there were any questions while directing their attention to the attending physician or the resident physician.

Additional data points were added to the survey to further blind the observers to the nature of the study. Data was entered into an electronic database by one of the study investigators (KS), and the attending and resident physicians were both coded as “male” or “female” based on their known gender identity.

Outcomes and data analysis

The primary outcome was the association between gender and to whom the paramedic’s handoff report was first directed. Secondary outcomes included the association of gender, to whom the EMS personnel’s handoff report was directed most, and whether EMS asked both resident and attending physicians if they had any questions based on gender. The data were analyzed using a chi-squared analysis to determine the association between the physician’s gender and each of the four outcomes. Physician gender was based on four attending-resident groupings: female-female, female-male, male-female, and male-male.

## Results

A total of 784 EMS handoff encounters were observed over the two-month data collection period. We excluded encounters in which it was uncertain to whom EMS personnel gave first attention (n = 2), majority attention (n = 4), or whether they asked if the resident had any questions (n = 2) during their presentation. EMS personnel gave first attention and majority attention more frequently to residents than attendings, regardless of gender (Tables 1, 2). There was no significant association between the gender of physicians and which physician received first attention (χ2 {1, N = 782} = 0.9736, p = 0.3238) or majority attention during case presentation (χ2 {1, N = 780} = 1.9414, p = 0.1635).

**Table 1:** Direction of First Attention

	Female Physician	Male Physician
Attending	101 (27.75%)	103 (24.64%)
Resident	263 (72.25%)	315 (75.36%)

**Table 2:** Direction of Majority Attention

	Female Physician	Male Physician
Attending	97 (26.65%)	93 (22.36%)
Resident	267 (73.35%)	323 (77.64%)

Regardless of gender, EMS personnel asked attending EM physicians if they had questions less frequently than they asked residents if they had questions. There was no significant association between the gender of the residents and how frequently the EMS asked if they had questions (χ2 {1, N = 782} = 0.3723, p = 0.5418). However, EMS personnel were more likely to ask male attendings if they had questions than female attendings (χ2 {1, N = 784} = 4.4319, p = 0.0353) (Tables 3, 4).

**Table 3:** EMS Personnel Asking if Provider Had Questions: Attending EMS: Emergency Medical Services

	Female Attending	Male Attending
Did Not Ask if Attending Had Questions	324 (88.77%)	350 (83.53%)
Asked if Attending Had Questions	41 (11.23%)	69 (16.47%)

**Table 4:** EMS Provider Asking if Provider Had Questions: Resident EMS: Emergency Medical Services

	Female Resident	Male Resident
Did Not Ask if Resident Had Questions	291 (79.73%)	325 (77.94%)
Asked if Resident Had Questions	74 (20.27%)	92 (22.06%)

## Discussion

Gender bias can have a large negative impact on female physicians [3]. Research on this topic has identified perceptions of gender bias through evaluation rather than directly observing gender bias [4,5]. Importantly, perceptions may not equate to direct observation of gender bias. To our knowledge, this study is the first in which direct observations between prehospital clinicians and physicians attempted to identify gender bias during patient handoffs. Direct observation of gender bias in the ED would identify opportunities for additional training and education to eliminate gender bias and microaggressions. 

Based on existing literature regarding the perception of females experiencing gender bias and our personal experiences in the ED, we hypothesized that EMS would more frequently focus their communication on male physicians. However, we found limited evidence to suggest gender bias exists during patient handoff. There was no significant association between the gender of physicians and which physician received the first or most attention. 

On the other hand, EMS personnel were more likely to address questions to male attendings than female attendings, possibly indicating gender bias. Interestingly, EMS personnel directed attention more frequently to the resident physicians during patient handoffs. This may be because residents at our institution are expected to assume the role of primary provider and immediately present themselves in that manner, prompting the EMS personnel to direct attention to the resident. 

Given the established existence of gender bias in medicine, it was reassuring that we saw limited evidence of such bias in this study. Additional research is needed to understand factors that impact a female physician’s perception of microaggressions and why the frequency of perceived gender bias may be higher than that of observed instances of gender bias.

This study has several limitations. First, there is inherent subjectivity when using observers for data collection. We tried to limit this subjectivity by training observers. Additionally, this study was done at a single academic medical center, and the emphasis on resident autonomy could have influenced the focus of EMS reports. We did not account for the gender of the paramedic, which could impact handoff interactions since females are known to be underrepresented in prehospital medicine [15]. Several additional variables that may have influenced a paramedic’s decision to report to one physician over another, such as proximity of physicians, body language, or seniority, were not assessed. Lastly, the metrics measured in this study may not capture the gender bias that female physicians have previously reported. The perception of the female physicians in this study was not recorded, and therefore it is possible that female physicians were experiencing microaggressions that weren’t necessarily related to the metrics we recorded.

## Conclusions

We identified limited differences in communication from paramedics to physicians during emergency department patient handoffs. In our study, we found no significant differences in how EMS personnel directed their attention based on gender. They were more likely to ask male attendings if they had questions than they were for female attendings, but this difference was not present among residents. Further research would be required to detect any physician perceptions of microaggressions during patient handoffs.
